# Macroporous polymer supported azide and nanocopper (I): efficient and reusable reagent and catalyst for multicomponent click synthesis of 1,4-disubstituted-1*H*-1,2,3-triazoles from benzyl halides

**DOI:** 10.1186/2193-1801-2-64

**Published:** 2013-02-23

**Authors:** Mosadegh Keshavarz, Nasir Iravani, Abdolmohammad Ghaedi, Amanollah Zarei Ahmady, Masoumeh Vafaei-Nezhad, Sara Karimi

**Affiliations:** 1 Department of Chemistry, Yasouj University, P.O. Box 353, Yasouj, 75918-74831 Iran; 2 Department of Chemistry, Gachsaran branch, Islamic Azad University, Gachsaran, Iran; 3 Medicinal Herbs and Natural Compounds researches Center, Jundishapur University of Medical Sciences, Ahvaz, Iran

**Keywords:** Polymer supported catalyst, Sodium azide, Triazole, Click chemistry, Benzyl halide

## Abstract

Macroporous polymer supported nanoparticles of copper(I) iodide catalyst and macroporous polymer supported azide reagent were used to simplify the synthesis of 1,4-disubstituted-1*H*-1,2,3-triazoles from various benzyl halides following the green chemistry principles. This new one-pot protocol facilitates the workup of the reaction and provides the products in short times and at high yields. Heterogeneous catalyst and reagent can be reloaded and reused at least for 5 runs without significant decrease in the yields.

**Electronic supplementary material:**

The online version of this article (doi:10.1186/2193-1801-2-64) contains supplementary material, which is available to authorized users.

## Background

Consistent with one of the basic principles of Green Chemistry, catalytic or recyclable reagents are better than stoichiometric reagents (Anastas and Warner 1998). Polymer-supported reagents have more and more attracted attention as insoluble matrices in organic synthesis (Kirschning et al. 2001; Hodge et al. 2005). They recommend rewards such as reaction monitoring as well as improved safety, more than ever when the non-supported reagents are toxic or unsafe as they can be easily removed from reaction medium and recycled (Galaffu et al. 2005; Erb et al. 2003). Additionally, employing an excess amount of reagent is permitted without the need for further purification.

Sodium azide is vital reagent and copper(I) is a wonderful catalyst for 1,3-dipolar cycloaddition between organic azides and terminal alkynes which is best known as click reaction (Lwowsky 1984; Rostovtsev et al. 2002; Hein et al. 2008). This cycloaddition has been used in various ways in drug discovery, chemical biology and medicinal chemistry (Moorhouse et al. 2008; Moorhouse and Moses 2008; Gil MV et al. 2007; Moses and Moorhouse 2007), as well as material science and solid phase organic synthesis (Yao et al. 2008; Wu et al. 2004; Bodine et al. 2004; Lober and Gmeiner 2004). Nevertheless, sodium azide and copper salts are highly toxic compounds. Sodium azide is a potent toxin, has a comparable toxicity as that of cyanide ion (LD_50_ = 27 mgkg^-1^ for rats) and can be absorbed through the skin mucous membranes. The excess amount of sodium azide is wasted during the nucleophilic substitution reaction in organic processes and pollutes the environment since it is not usually recovered from reaction medium.

Despite of toxicity, copper(I) salts have been less used as catalyst for this cyclization in view of the fact that they are thermodynamically instable and the formation of undesired alkyne-alkyne coupling products which is sometimes observed in their existence (Tornøe et al. 2002; Aucagne and Leigh 2006). On the other hand, these homogeneous processes suffer from one or more disadvantages such as difficulty in separation of the product from the reaction medium. These homogenous catalysts are usually non-recyclable, have low regioselectivity and long reaction times. However, nitrogen or phosphorus-based ligands have been shown to protect the metal center from oxidation and disproportionation, while enhancing its catalytic activity (Gerard et al. 2006; Marra et al. 2008; Broggi et al. 2008). Recently, heterogeneous copper(I) catalysts based on silica (Miao and Wang 2008; Shamim and Paul 2010), montmorillonite (Jlalia et al. 2008), zeolites (Bénéteau et al. 2010; Chassaing 2010) and Cu in charcoal (Sharghi et al. 2009) and Cu nanoporous skeleton catalyst (Jin et al. 2011, 2012; Asao et al. 2012) have been developed. There are also some reports on using polymer supported CuI as recoverable catalyst for this cyclization (Albadi et al. 2012a, 2012b; Albadi and Keshavarz 2013; Girard et al. 2006; Jlalia et al. 2010; Dervaux and Du Prez 2012).

In recent years, nano-catalysis has emerged as a sustainable and competitive alternative to conventional catalysis since the nanoparticles possess a high surface-to-volume ratio, which enhances their activity and selectivity, while at the same time maintaining the intrinsic features of a heterogeneous catalyst (Astruc 2008). In particular, the immobilization of copper (I) salts nanoparticles on high-surface-area supports allows a higher stability and dispersity of the particles as well as a further exploitation of the special activity and recycling properties of the catalyst. These catalysts possess better advantages than their homogeneous counterparts. However, to the best of our knowledge, there is no report for one-pot multicomponent synthesis of 1,4-disubstituted-1*H*-1,2,3-triazoles which utilize polymer supported azide which can significantly reduce the toxicity and ecological impacts of this reagent. Herein we report a new, facile and green procedure for on-pot multicomponent click synthesis of 1,4-disubstituted-1*H*-1,2,3-triazoles from benzyl halides and terminal alkynes using polymer supported azide and nanoparticles of CuI.

## Experimental section

### Materials and methods

All of the triazole derivatives were prepared by our procedure; their spectroscopic and physical data were compared with those of authentic samples. NMR spectra were recorded in DMSO-d_6_ or CDCl_3_ on a Bruker Advanced DPX 500 and 400 MHz instrument spectrometers using TMS as internal standard. IR spectra were recorded on a BOMEMMB-Series 1998 FT-IR spectrometer.

#### Preparation of polymer-supported azide reagent (IRA-910 N_3_)

Amberlite *IRA-400* N_3_ form was easily prepared from its corresponding chloride form via ion exchange using 10% NaN_3_ aqueous solution. Two grams of amberlite *IRA-400* chloride form (mesh 16–50) were stirred for 6 h in the corresponding solution (100 mL of 10% NaN_3_ aqueous solution), filtered-off and washed several times with water and stirred for additional 5 min, it was then decanted, washed several times with water until the supernatant liquid gave a negative azide test with ferric nitrate and dried under vacuum at 50°C.

The exchange capacity of the resin was determined by passing 1 N sodium chloride solution (50 mL) through the resin (0.3 g) packed in a column. The amount of sodium salt of the nucleophile in the eluent was then titrated with 0.01 N hydrochloric acid using methyl orange as indicator. The exchange capacity of polymer supported nucleophile was calculated to be 3.5 mmolg^-1^ of N_3_^-^.

#### Preparation of the supported catalyst (A-21CuI)

CuI (100 mg) was dissolved in 30 mL absolute ethanol, and magnetically stirred in a pre-heated oil bath at reflux temperature for 4 h under a nitrogen atmosphere in the presence of dry amberlyst *A-21* (1.0 g, 4.8 mmol amine; mesh 20–50). The resulting materials were washed with ethanol (4 × 30 mL) and dried under vacuum at 40°C overnight. To evaluate the copper content, the A21-Cu (100 mg) was extracted with concentrated HCl (5 × 2 mL) in a screw-capped vessel, followed by treatment with concentrated nitric acid (2 mL) to digest the metal complex. The mixture was then transferred into a volumetric flask (100 mL), diluted 1:50 for the second time and was analyzed by the ICP analysis. The copper concentration was determined from the atomic emissions (324.754 nm) by reference to a linear (R = 0.99) calibration curve of (1–4 ppm) CuI prepared in a manner identical to the sample preparation. The copper content of *A21-CuI* was calculated to be 11.7% w/w.

Typical procedure for multicomponent synthesis of 1-phenyl-2-(4-phenyl-1-H-1,2,3-triazol-1yl)-1-ethanone (Table 1, entry 1).

**Table 1:** **Synthesis of 1,4-disubstituted-1*H*-1,2,3-triazoles using polymer supported reagent and catalyst**

Entry	Benzyl halide (a)	Product (b)	Time	Yield	m.p./Lit. m.p °C
			(h)	(℅)^c^	
**1**			1	92	128-129/128-129.5 (Shamim and Paul 2010)
**2**			1	89	
**3**			1.5	83	110/ 109–110 (Sharghi et al. 2009)
**4**			1.5	80	117-119
**5**			1.5	79	140–141/ 140–142 (Shamim and Paul 2010)
**6**			1.5	85	147-149
**7**			1.5	87	152-152.5/ 151–152 (Sharghi et al. 2009)
**8**			1.5	75	190-192/ 191 (Sharghi et al. 2009)
**9**			2	79	108-110/110 (Sharghi et al. 2009)
**10**			1.5	83	196-198/ 197 (Sharghi et al. 2009)
**11**			1.5	85	197 (Sharghi et al. 2009)
**12**			1.5	81	139-141
**13**			1	74	137-139
**14**			1.5	70	130-131
**15**			1.5	79	131-133

^c^ Yields refer to isolated and pure products.

Benzyl halide (1 mmol, 2 mmol for entries 13 and 14) and 1,3-diethynylbenzene (1 mmol) were placed together in a round-bottom flask containing 10 mL of ethanol. Amberlyst *A-21*CuI (1 mo%, 0.03 g) and amberlite *IRA-910* N_3_ (0.5 g, loading: 3.5 meqg^-1^) were added at once to the mixture. The suspension was magnetically stirred under reflux conditions for appropriate time shown in Table 1. After completion of the reaction as followed by TLC (n-hexane: ethyl acetate; 4:1), the resins were filtered and washed with hot ethanol (2 × 5 mL). The filtrates were evaporated to dryness, and then the solid residue was recrystallized in ethanol/water (1:3 v/v) to give pure product crystals.

#### Reusing of polymer supported catalyst and reagent

The filtered mixture of resins was washed with 25 mL of distillated water and stirred for 6 h in the corresponding solution (50 mL of 10% NaN_3_ aqueous solution) and dried under vacuum at 50°C before next run.

#### Selected spectral data

*1-(4-methoxybenzyl)-4-phenyl-1H-1,2,3-triazole* (Table 1, 3b): m.p 128–130; ^1^H NMR (CDCl_3_, 500 MHz) δ / ppm : 7.81 (d, 2H, *J =* 11 Hz), 7.71 (s, 1H), 7.40 (t, 2H, *J =* 8.4 Hz), 7.34-7.29(m, 2H), 6.91-6.84(m, 3H), 5.53(s, 2H), 3.78(s, 3H).

*1-(2,4-dichlorobenzyl)-4-phenyl-1H-1,2,3-triazole* (Table 1, 5b): m.p 147–149; ^1^H NMR (CDCl_3_, 500 MHz) δ / ppm : 7.86(d, 2H, *J = 9* Hz), 7.8(s, 1H), 7.52(d*,* 1H, *J = 2 Hz*), 7.46(t, 2H, *J =* 7.5 Hz), 7.38(t, 1H, *J =* 7.5 Hz), 7.32-7.30(m, 1H), 7.21(d, 1H, *J = 8* Hz), 5.72(s, 2H).

*1-(4-bromobenzyl)-4-phenyl-1H-1,2,3-triazole* (Table 1, 6b): m.p 152–152.5/151-152;^12^^1^H NMR (CDCl_3_, 400 MHz) δ / ppm : 7.82(d, 2H, *J =* 7.5 Hz), 7.69 (s, 1H), 7.54(d, 2H, *J =* 8.3 Hz), 7.43(t, 2H, *J =* 7.5 Hz), 7.35(t, 1H, *J =* 7.5 Hz), 7.21(d, 2H, *J =* 8.3 Hz), 5.56(s, 2H).

*3-(1-benzyl-1H-1,2,3-triazole-4-yl)aniline* (Table 1, 12b): IR (KBr): 3420 and 3338(NH_2_) cm^-1^, ^1^H-NMR(500 MHz, DMSO-d_6_): δ /ppm : 8.35 (s, 1H), 8.11 (d, 2H, *J* = 7.46 Hz), 7.75 (t, 1H, *J* = 7 Hz), 7.63 (t, 2H, *J* = 7.32 Hz), 7.15 (s, 1H), 7.9 (t, 1H, *J* 7.6 Hz), 6.98 (d, 1H, *J* = 7.5 Hz), 6.56 (d, 1H, *J* = 7.5Hz), 6.22 (s, 2H), 5.22 (s, 2H); ^13^C NMR (125 MHz, DMSO-d_6_): δ / ppm : 149.9, 147.85, 135.13, 135.02, 132.09, 130.27, 129.88, 129.8, 123.46, 114.48, 113.9, 111.38, 56.78.

*1-(2,4-dichlorobenzyl)-4-{3-[1-(2,4-dichlorobenzyl)-1H-1,2,3-triazole-4-yl]phenyl}-1H-1,2,3-triazole* (Table 1, 14b): m.p 131–133. ^1^H NMR (DMSO-d_6_, 400 MHz) δ / ppm : 8.67(s, 2H), 8.34(s, 1H), 7.82(d, 2H, *J =* 7.7 Hz), 7.73(d, 2H, *J =* 1.8 Hz), 7.51-7.49(m, 3H), 7.36(d, 2H, *J = 8.3* Hz), 5.76(s, 4H); ^13^C NMR (100 MHz, DMSO-d_6_) δ / ppm : 146.7, 134.5, 134.3, 132.7, 132.5, 131.6, 130, 129.6, 128.4, 125.2, 122.6, 122.3, 50.8.

## Results and discussion

**Figure 1:**
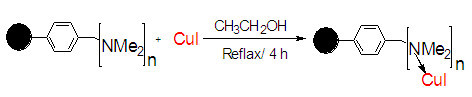
**Preparation of Amberlyst supported nanoparticles of CuI (*A-21*CuI).**

**Figure 2:**
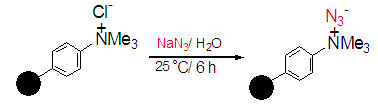
**Preparation of amberlite supported azide (*IRA-400*** **N**_**3**_**).**

This situation requires the development of a novel polymer-supported heterogeneous catalyst and reagent that can join the advantages of both, homogenous and heterogeneous in order to obtain competent reagent and catalyst. The preparation procedures followed to obtain macroporous polymer supported nanoparticles of CuI catalyst and polymer supported azide reagent are outlined in Figures 1 and 2. These consist of building up suitable heterogeneous polymer supported nanocopper (I) catalyst (Figure 1) and macroporous polymer supported azide nucleophile (Figure 2) structures on the surface of commercial available amberlyst *A21* (mesh 20–50) and amberlite *IRA-400*Cl (mesh 16–50). Preparation of heterogeneous copper(I) iodide catalyst and polymer supported azide reagent by these procedures are facile and straightforward.

**Figure 3:**
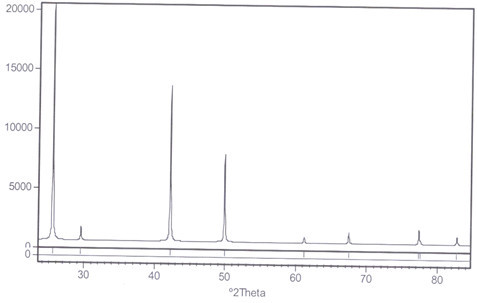
**XRD pattern of amberlyst supported CuI.**

**Figure 4:**
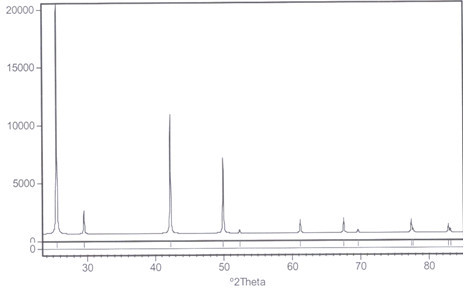
**XRD pattern of pure CuI.**

The nanocopper catalyst immobilized on amberlyst A21 (mesh 20–50) was readily prepared in a one-step procedure. Amberlyst A21 was refluxed with CuI under an N_2_ atmosphere in EtOH to afford the polymer-supported CuI nanoparticles catalyst. Immobilization of nanoparticles of CuI on amberlyst support (A21-CuI) was approved by scanning electron microscopy (SEM, see the Additional file 1), IR, X-ray diffraction (XRD) and ICP analysis techniques. The SEM image of the prepared catalyst indicated that CuI nanoparticles were homogeneously immobilized on amberlyst surface. According to the SEM images of the obtained A21-CuI, the average size of copper nanostructures was estimated to be 75–90 nm. The sharp peaks of copper were observed in the XRD patterns of copper iodide on amberlyst (Figure 3). Their positions were consistent with metallic copper (Figure 4, also see the Additional file 1) and copper iodide nanocrystals and confirmed the presence of copper iodide on the amberlyst surface again. The size of copper nanoparticle was also determined from X-ray line broading using the Debye–Scherrer formula (obtained size: 75 nm).

Amberlite *IRA-400* N_3_ form was easily prepared from its related chloride form via ion exchange using 10% NaN_3_ aqueous solution. Appearing a signal in 2050 cm^-1^ region in the IR spectrum of IRA400N_3_ confirmed the presence of azide on the surface on this commercial available ion exchange polymer. The exchange capacity of the IRA400N_3_ was determined by passing 1 N sodium chloride solution through the IRA400N_3_ packed in a column. The amount of sodium salt of the nucleophile in the eluent was then titrated with 0.01 normal hydrochloric acid using methyl orange as indicator. The exchange capacity of polymer supported nucleophile was calculated to be 3.5 mmolg^-1^ of N_3_^-^.

After preparation of polymer supported catalyst and reagent we began to optimize the reaction conditions. Phenyl acetylene and benzyl bromide were selected as the test substrates and acetonitrile, methylene chloride, ethanol and water as solvents. Unluckily, we did not get good results at room temperature with various amounts of heterogeneous catalyst and reagent with each of solvents after 4 hours. So the reaction was followed at reflux conditions.

**Figure 5:**
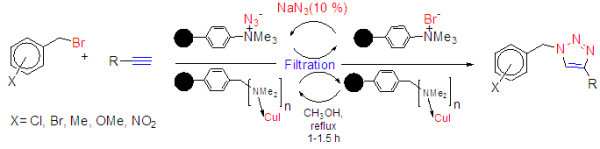
**Multicomponent synthesis of 1,4-disubstituted-1H-1,2,3- triazoles using recoverable polymer supported azide and CuI.**

From Table 2, ethanol, *A-21CuI* (60 mg, 10℅) and *IRA-400 N*_*3*_ (0.5 g) are optimized conditions for multicomponent click cyclization. The results were evaluated qualitatively through TLC (Table 2). The best conditions employ 1:1:1.5:0.1 mol ratios of phenyl acetylene, benzyl bromide, *IRA-400 N*_*3*_ and *A-21CuI* at reflux conditions using ethanol as solvent. Using these optimized conditions, the reaction of various terminal alkynes and benzyl bromide was examined (Figure 5).

**Table 2:** **The effect of solvent on the reaction progress**

Entry	Solvent	***IRA-400*** N_3_	***A-21CuI***	Time (h)	Yield (℅)^a^
1	CH_2_Cl_2_	0.5 g	60 mg	2	41
2	H_2_O	0.5 g	60 mg	2	39
3	CH_3_CH_2_OH	0.5 g	60 mg	1	90
4	CH_3_CN	0.5 g	60 mg	1	83

^a^ Yield refer to pure and separated products.

All the products were cleanly isolated with simple filtration and evaporation of solvent (Table 1). The solid products were easily recrystallized from a mixture of ethanol/water (1:3 v/v) or in some cases from ethanol (Table 1, entry 13 and 14). Click condensations were confirmed by the appearance of a singlet in the region of 8–8.5 ppm in ^1^H-NMR spectra, which corresponds to the hydrogen on 5-position of triazole ring and confirms the regioselective synthesis of 1,4-disubstituted triazole regioisomers (Keshavarz and Badri 2011).

**Figure 6:**
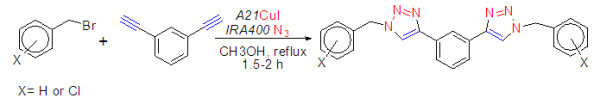
**Synthesis of symmetrical substituted bis-triazoles using 1,3-diethynylbenzene.**

Since organic azides are often unstable to heat and light, their in situ preparation recommends a great choice to their use and handling. All benzyl azides were prepared in situ and subjected to multicomponent click cyclization with various terminal alkynes. The using of 1,3-diethynylbenzene in click cyclizations led to the synthesis of some interesting symmetrical bis-triazoles (Table 1, entry 13, 14 and Figure 6).

The recyclability and reusability of the supported catalyst or reagent is important. To investigate this property for our introduced catalyst, the reaction of benzyl bromide with phenyl acetylene was selected again as model (Table 3).

**Table 3:** **Recyclability and reusability of polymer supported azide and CuI**

Entry	1	2	3	4	5
Number of loading	1	2	3	4	5
Yield (%)^a^	90	90	89	87	87

^a^ Yields refer to isolated and pure products.

After reaction completion, the resins mixture was washed with distilled water and then subjected to aqueous NaN_3_ solution to reload *IRA-400* N_3_. This process repeated for five runs and no appreciable yield decrease was observed. Almost consistent activity was observed over five runs (Table 3).

Next we checked the leaching of CuI nanoparticles into the reaction mixture from the amberlyst support using ICP-AES. The difference between the copper content of the fresh catalyst and the used catalyst (5th run) was only 3% which indicated the low leaching amount of copper iodide catalyst into the reaction mixture.

The results suggest that the catalyst and reagent developed are maintaining their efficiency in repeated uses. Polymer supported CuI can be reused up to five runs without need to reload and Polymer supported azide nucleophile can be loaded several times.

Table 4 represents the efficiency of the introduced method in comparison with some of the reported methodologies. Although in some previous reports the rate of the reaction is faster than our present method (entries 2, 3 and 4), the superiority of this work is that both catalyst and reagent (N_3_^-^) are recoverable. In is worth to note that the yield of the product by our procedure is comparable with reported methods

**Table 4:** **Evaluation of the introduced methodology in comparison with some of the previously reported methods**

Entry	Reagent/catalyst/ solvent	t (°C)	Time (h)	Yield	Reference
1	NaN_3_/ Silica-supported Cu(I)/ EtOH	78	24 h	93	(Miao and Wang 2008)
2	NaN_3_/ Silica-supported Cu(I)/ H_2_O	25	0.25	91	(Shamim and Paul 2010)
3	NaN_3_/ Nano Cu(I) on Charcol/ H_2_O	100	0.6	91	(Sharghi et al. 2009)
4	NaN_3_/ P_4_VPy-CuI / H_2_O	100	0.25	90	(Albadi et al. 2012b)
5	NaN_3_ / CuI/ i-Pr_2_EtN (additive) /[C_8_dabco][N(CN)_2_]	25	16	95	(Marra et al. 2008)
6	IRA-400 N_3_/ A-21CuI/ CH_3_CH_2_OH	80	1	92	(This work)

## Conclusions

In conclusion, we have developed a simple, benign and multicomponent regioselective synthesis of biologically important 1,4-disubstituted-1*H*-1,2,3-triazoles with short times in high yields under polymer supported catalyst and reagent conditions. The methodology is conveniently applicable to a wide range of benzyl halides and acetylenic compounds, and permits the assembly of a diverse set of 1,4-disubstituted-1*H*-1,2,3-triazoles. The final reaction product can be simply filtered and separated without the need for a further chromatographic step. In addition, the spent polymeric reagent can be regenerated and reused several times without appreciable loss in its capacity and efficiency. Using of *A-21*CuI as catalyst protects the metal center from oxidation and disproportionation, while enhancing its catalytic activity and makes it to be a reusable catalyst at least for five runs. Minimal waste generation of this “user-friendly” process should be beneficial for industrial applications.

## Authors' information

Dr. Mosadegh Keshavarz: He was born in 1980 in Dehdasht, Iran. He received a B.Sc. degree of pure chemistry from the Teacher Training University of Tehran and after studying for three years at the Shahid Chamran University of Ahvaz, he obtained a M.Sc. degree in Organic Chemistry under the supervision of Professor Rashid Badri. He received his Ph.D. degree of organic chemistry from Chamran University in 2012 and is currently working as assistant professor at Yasouj University.

Dr. Nasir Iravani: He finished his B.Sc and M.Sc degrees in Organic Chemistry from Islamic Azad University of Chachsaran. He received his Ph.D. of Organic Chemistry from Science and Research Campus, Islamic Azad University, Ponak, Tehran at 2010 and is currently working at Islamic Azad University of Gachsaran.

Dr. Amanollah Zarei Ahmady: He finished his B.Sc, M.Sc and Ph.D. degrees in Organic Chemistry from Shahid Chamran University of Ahvaz. He is currently working as assistant researcher in the Medicinal Herbs and Natural Compounds researches Center, Jondi Shapour University of Ahvaz.

Dr. Abdolmohammad Ghaedi: He finished his B.Sc degree of pure Chemistry from Shiraz University. He received his M.Sc degree in Applied Chemistry from Kermanshah University and his Ph.D. of Applied chemistry from Science and Research Campus, Islamic Azad University, Ponak, Tehran at 2011 and is currently working at Islamic Azad University of Gachsaran.

Miss Masoumeh Vafaeen-nezhad: She received her B.Sc degree of Chemistry degree of Pure Chemisry from Shahid Chamran University and her M.Sc degree of Organic Chemistry from Islamic Azad University of Gachsaran. She is continuing her Ph.D. theses at Islamic Azad University of Arak.

Miss Sara Karimi: She received her B.Sc degree of Chemistry degree of Applied Chemistry from Islamic Azad University of Gachsaran. She is continuing her M.Sc degree of Organic Chemistry at Persian Gulf University.

## Electronic supplementary material

Additional file 1: **SEM image of Amberlyst supported nanoparticles of CuI. XRD spectrum of Amberlyst supported nanoparticles of CuI.** IR spectrum of amberlite supported azide (*IRA-400*N_3_). (DOCX 4 MB)
