# Supplementary material for: Macroporous polymer supported azide and nanocopper (I): efficient and reusable reagent and catalyst for multicomponent click synthesis of 1,4-disubstituted-1H-1,2,3-triazoles from benzyl halides
Source: Springerplus. 2013 Feb 23;2:64. doi: 10.1186/2193-1801-2-64 (PMC3647102; doi:10.1186/2193-1801-2-64)

**Macroporous polymer supported azide and nanocopper (I): efficient green reusable reagent and catalyst for multicomponent click synthesis of 1,4-disubstituted-1H-1,2,3-triazoles from benzyl halides**

Mosadegh Keshavarz,* ^a,b^ Bahador Karami,^a^ Abdolhamid Ghaedi,^b^ Jalal Albadi,^b^ Hakimeh Vafaei,^b^ and Masoumeh Vafaei-nezhad

^a^ Yasouj University, Yasouj 75918-74831, Iran. Tel: 7412223048

^b^ Islamic Azad University, Gachsaran branch, Gachsaran, Iran

Email: chem.mosadegh@gmail.com

**Abstract**

Macroporous Polymer supported nanocopper(I) iodide catalyst and Macroporous polymer supported azide reagent were used to simplify the synthesis of 1,4-disubstituted-1H-1,2,3-triazoles from various benzyl halides following the green chemistry principles. This new one-pot protocol facilitates the workup of the reaction and provides the products in short times and at high yields. Heterogeneous catalyst and reagent can be reloaded and reused at least for 5 runs without significant decrease in the yields.

**Keywords:** Polymer supported catalyst, Sodium azide, triazole, Click chemistry, Benzyl halide





SEM image of Amberlyst supported nanoparticles of CuI


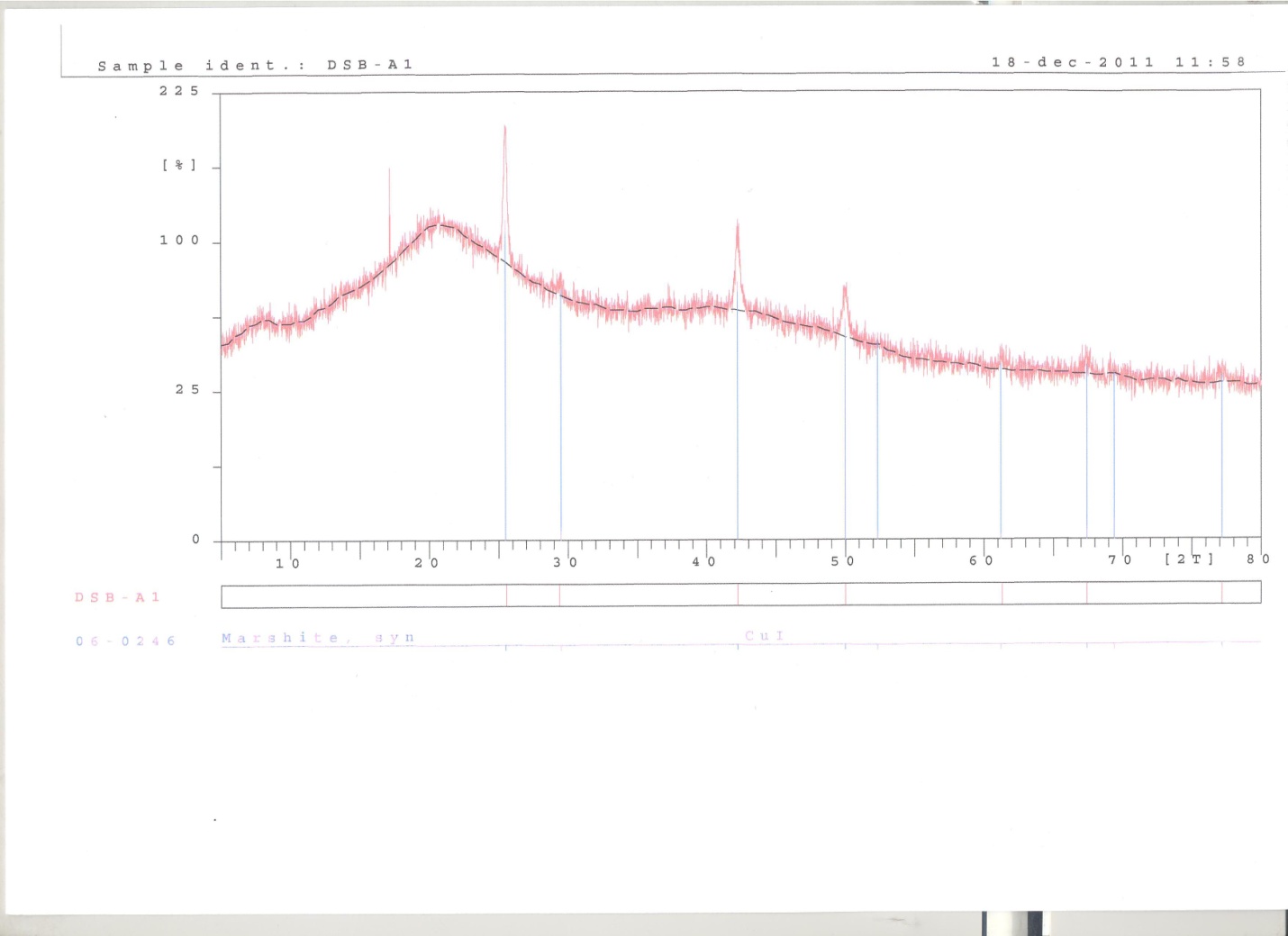


XRD spectrum of Amberlyst supported nanoparticles of CuI


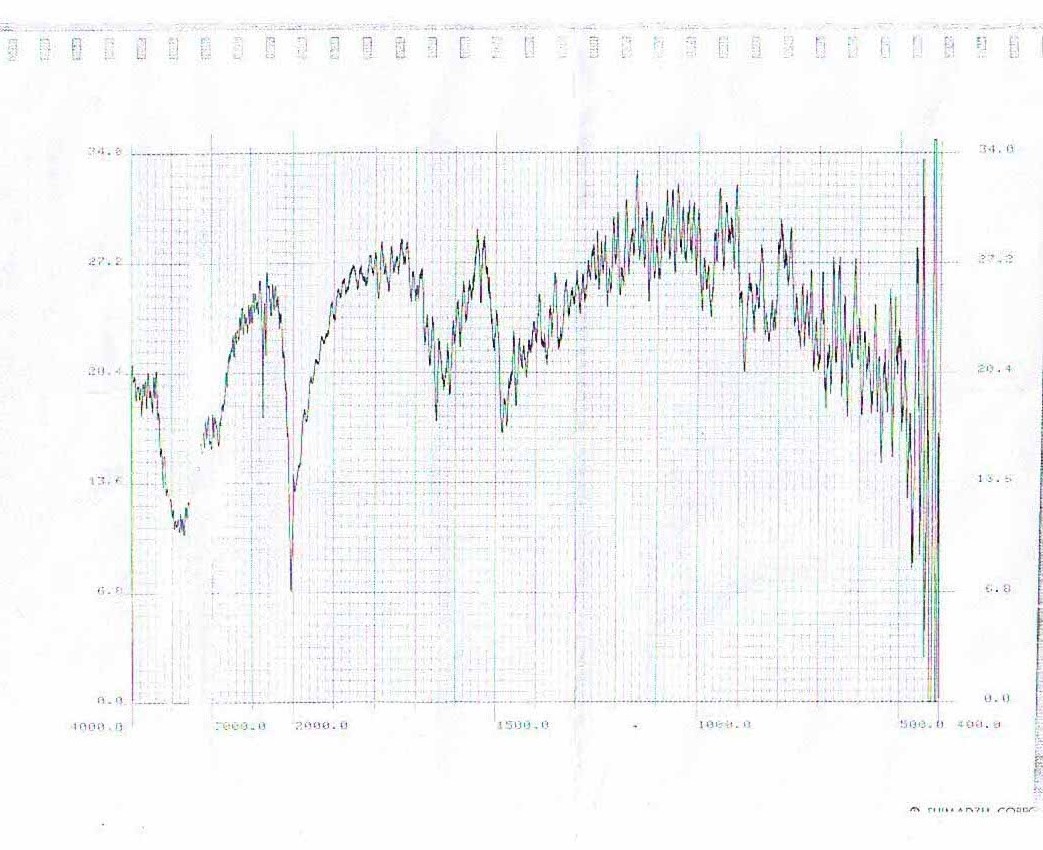


IR spectrum of amberlite supported azide (*IRA-400*N_3_)

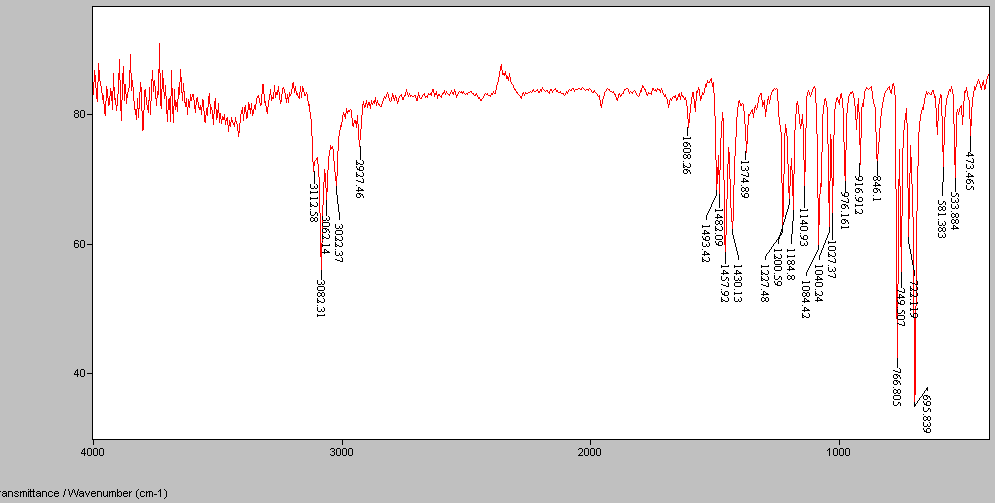

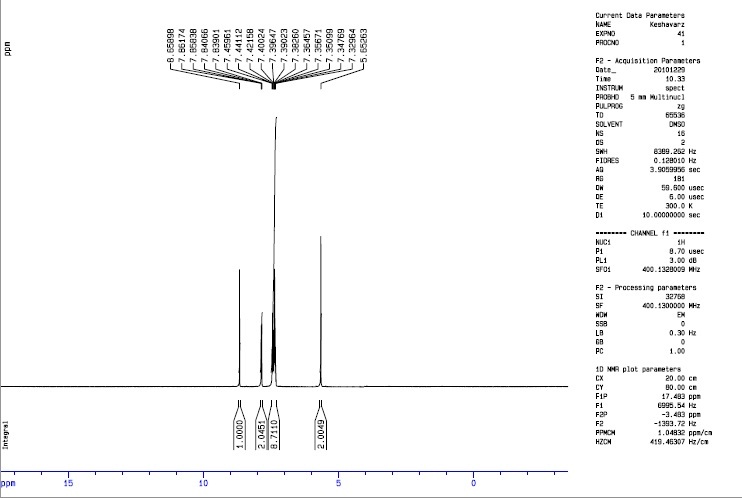

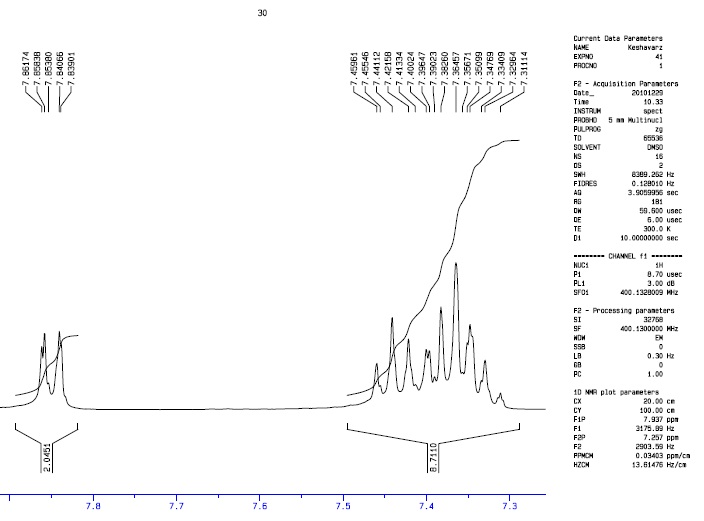

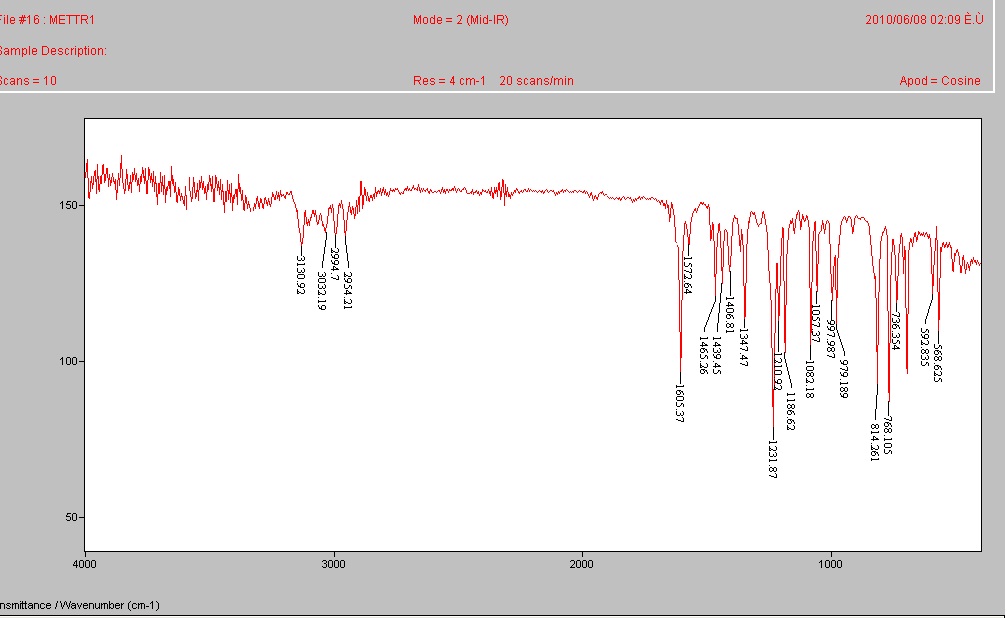

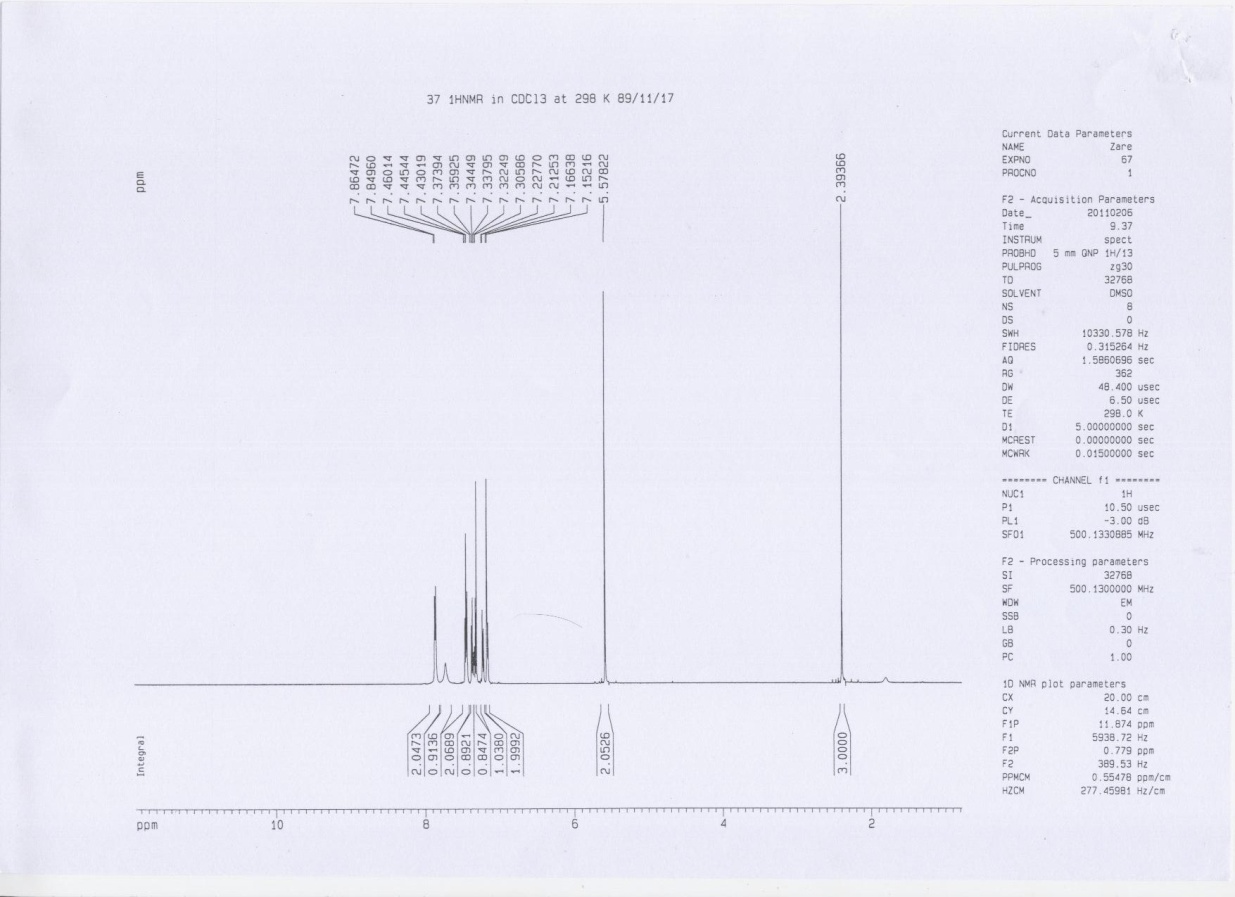

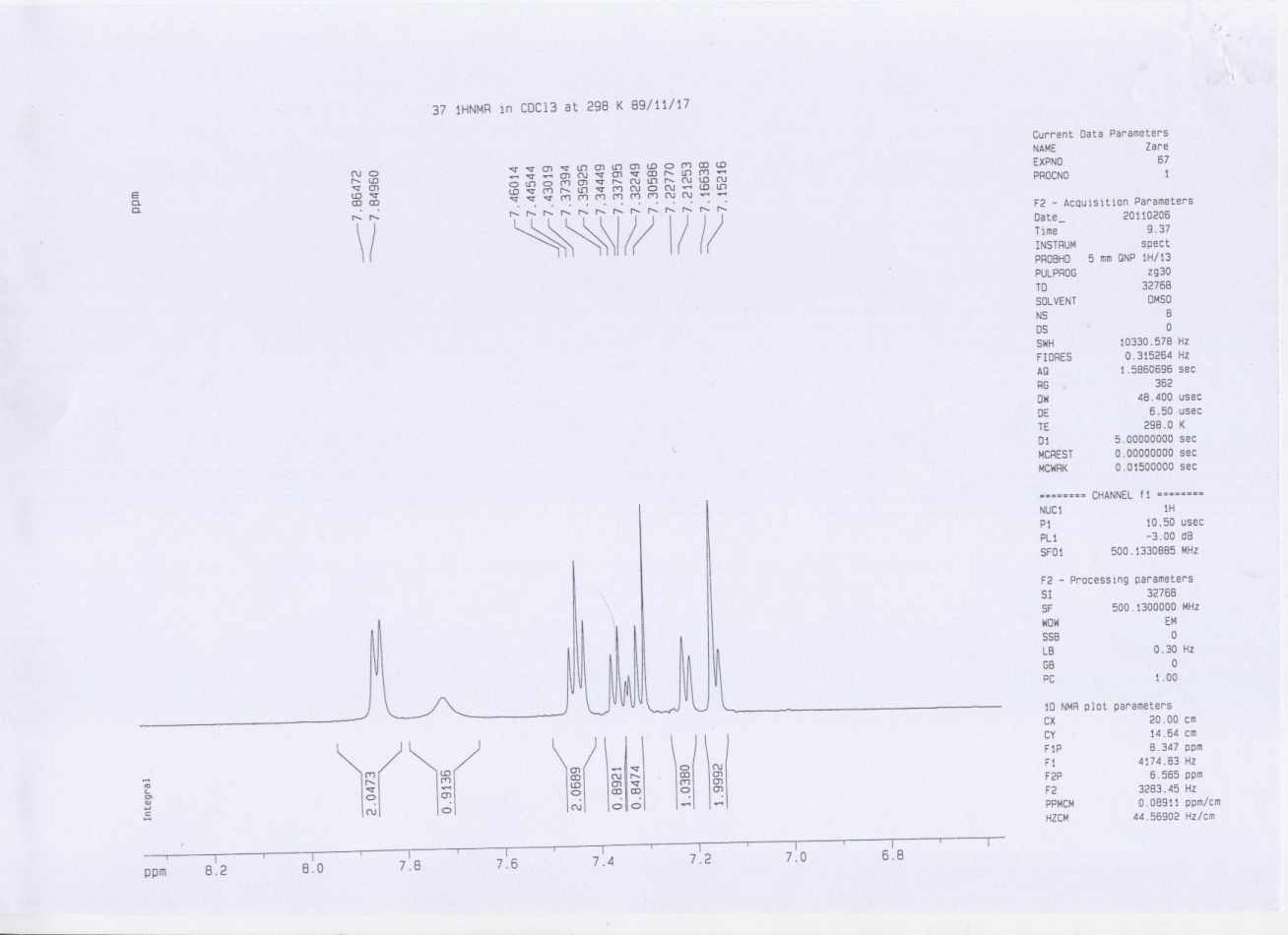

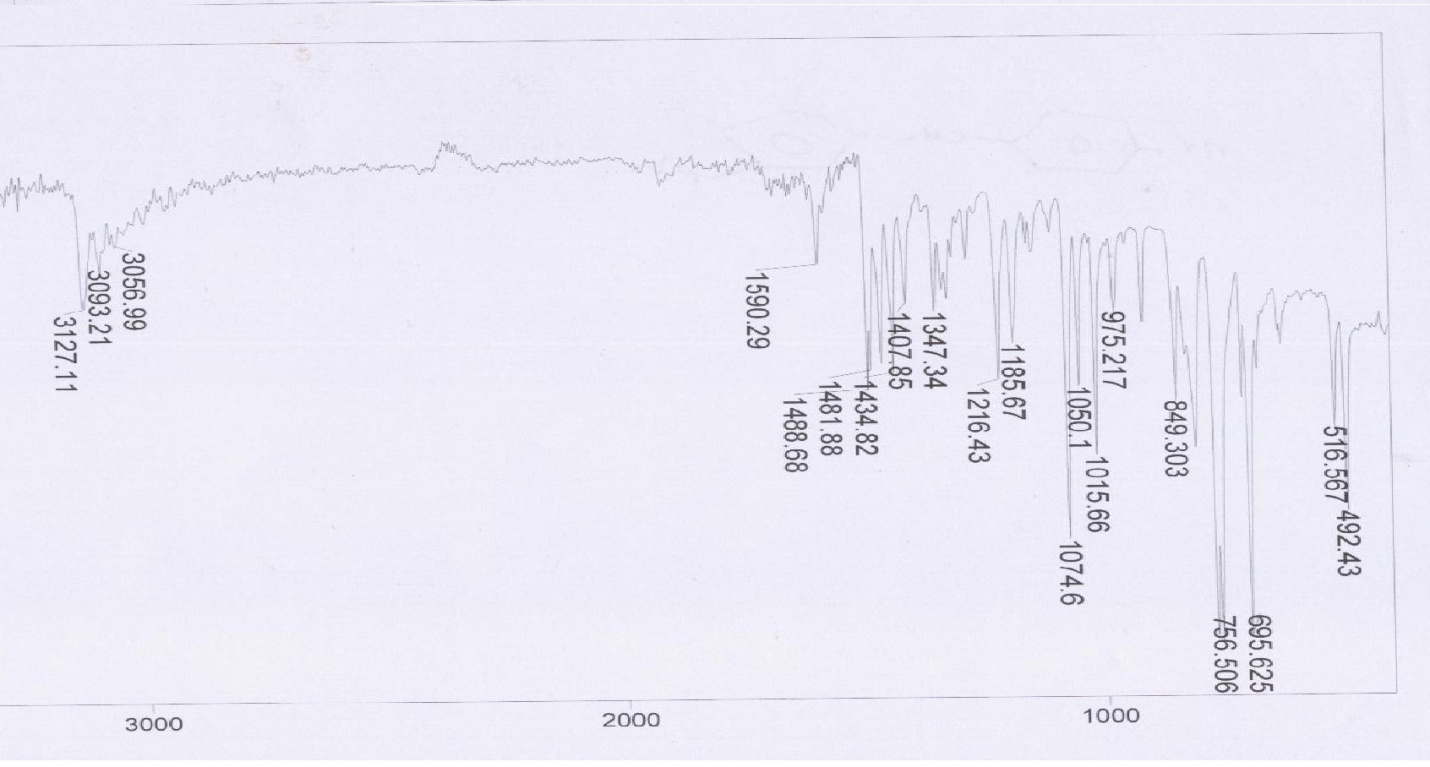

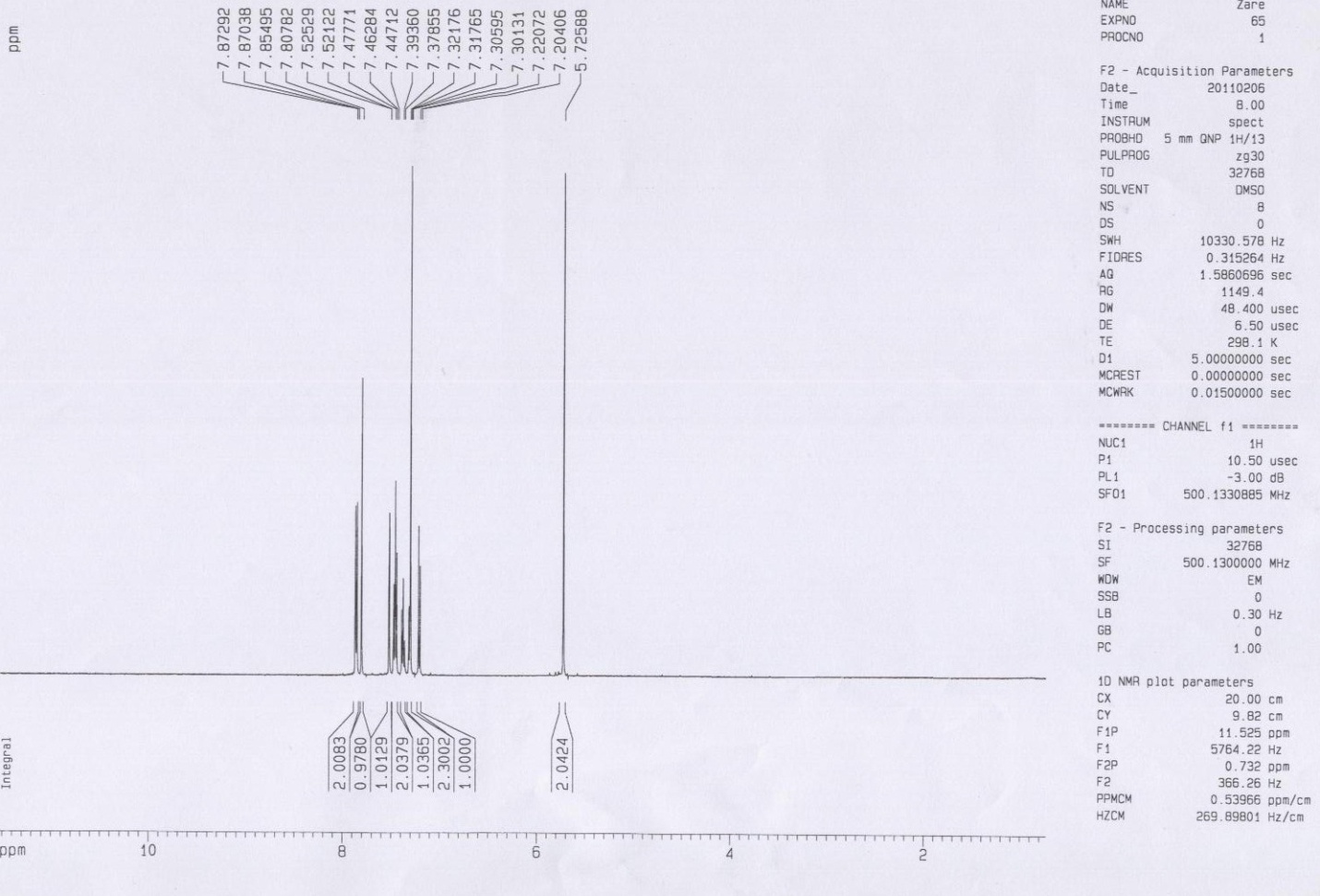

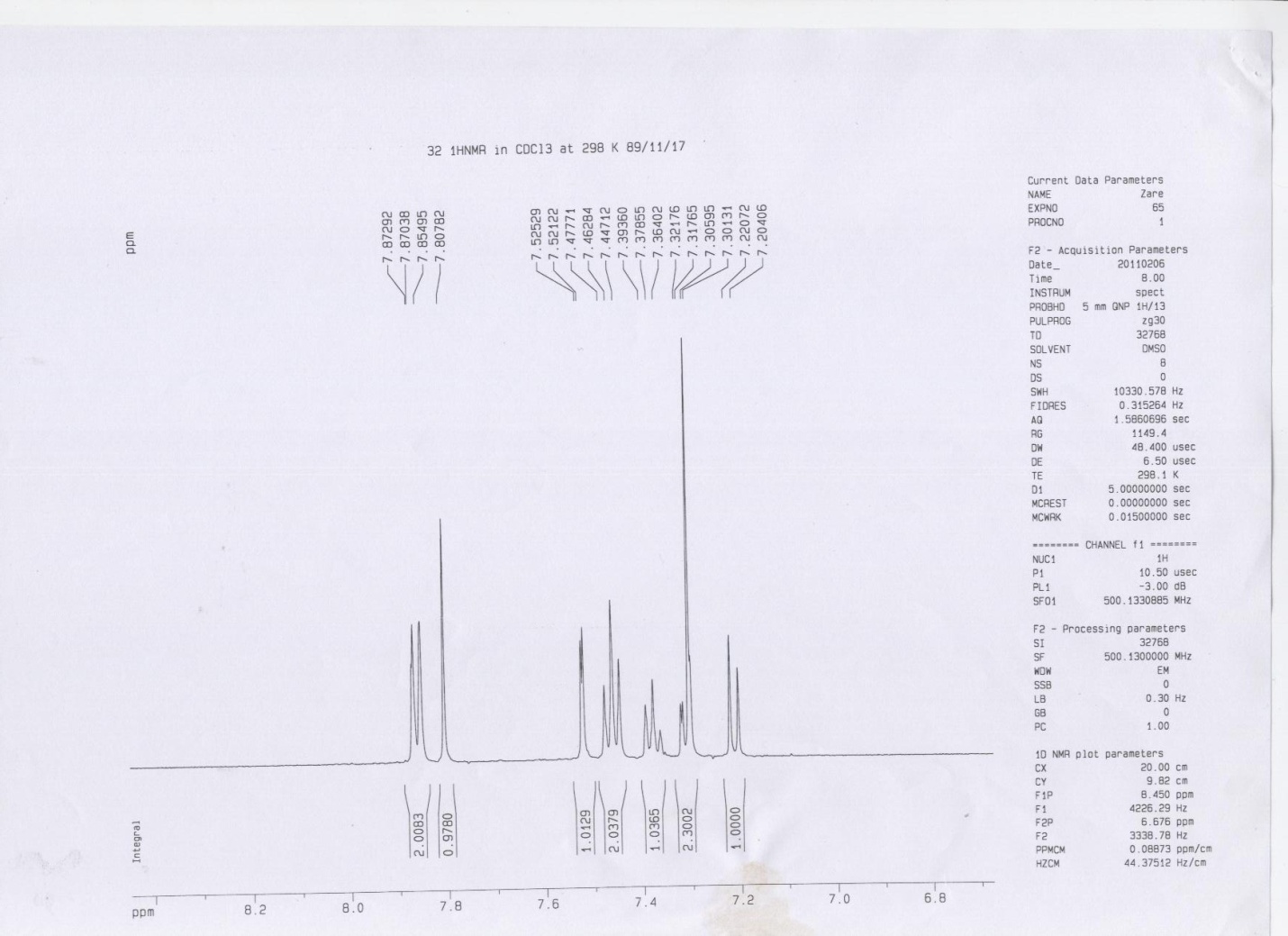

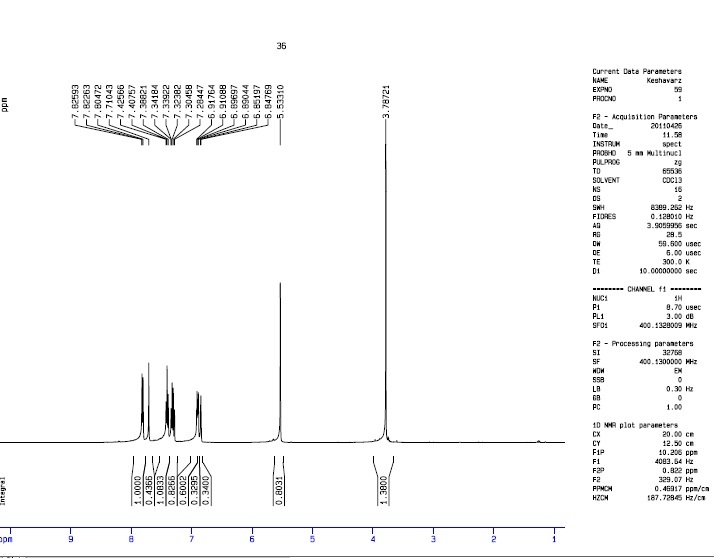

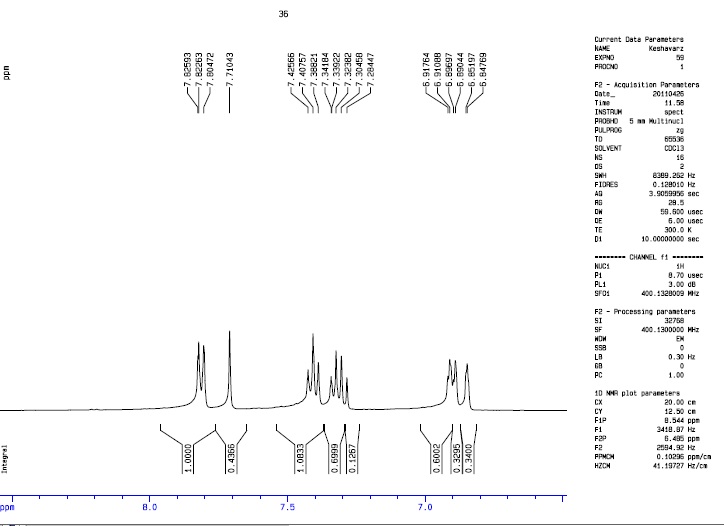

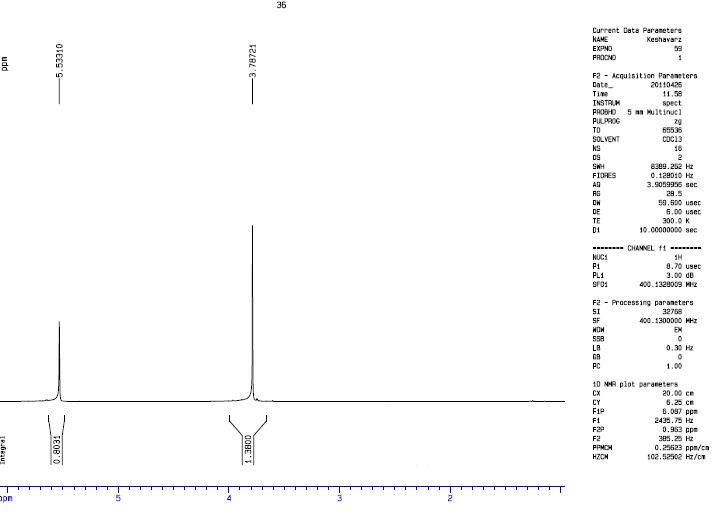

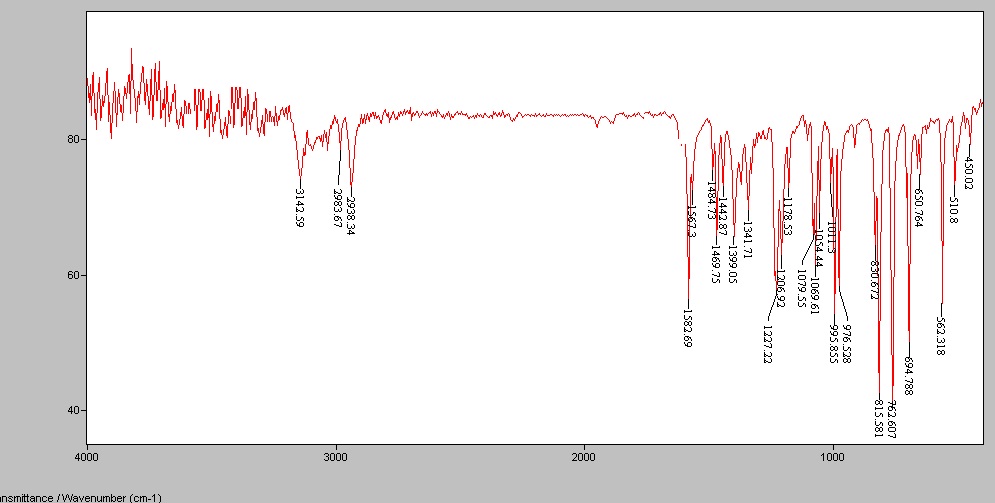

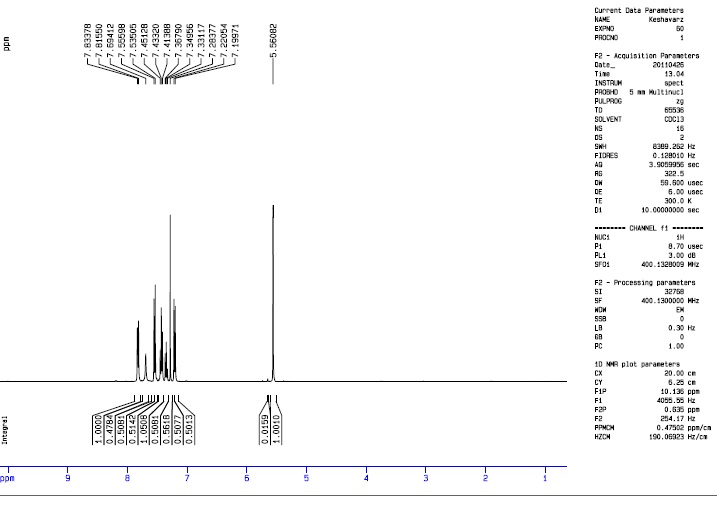

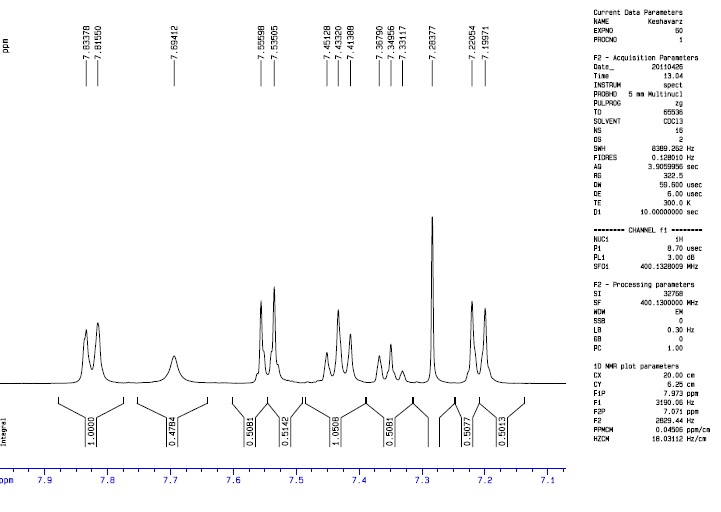

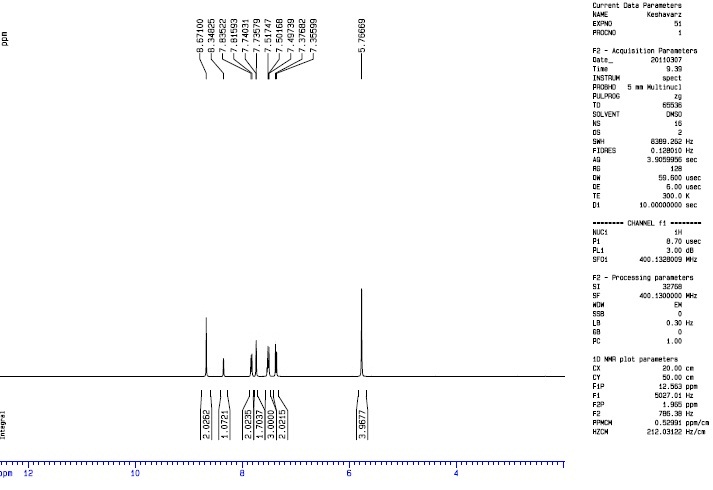

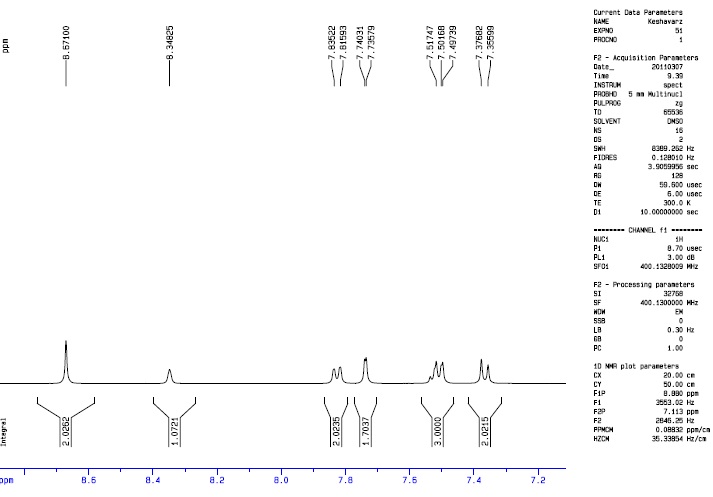

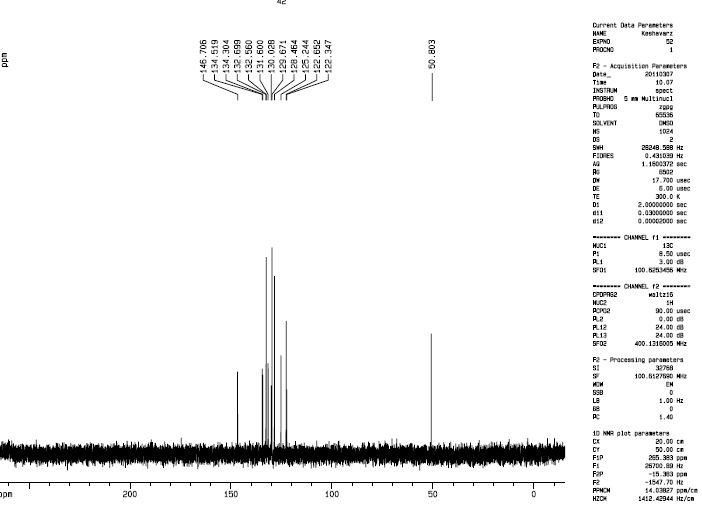

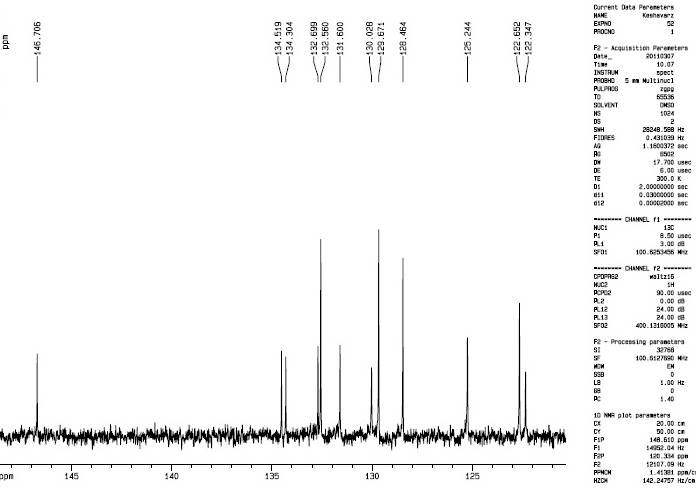

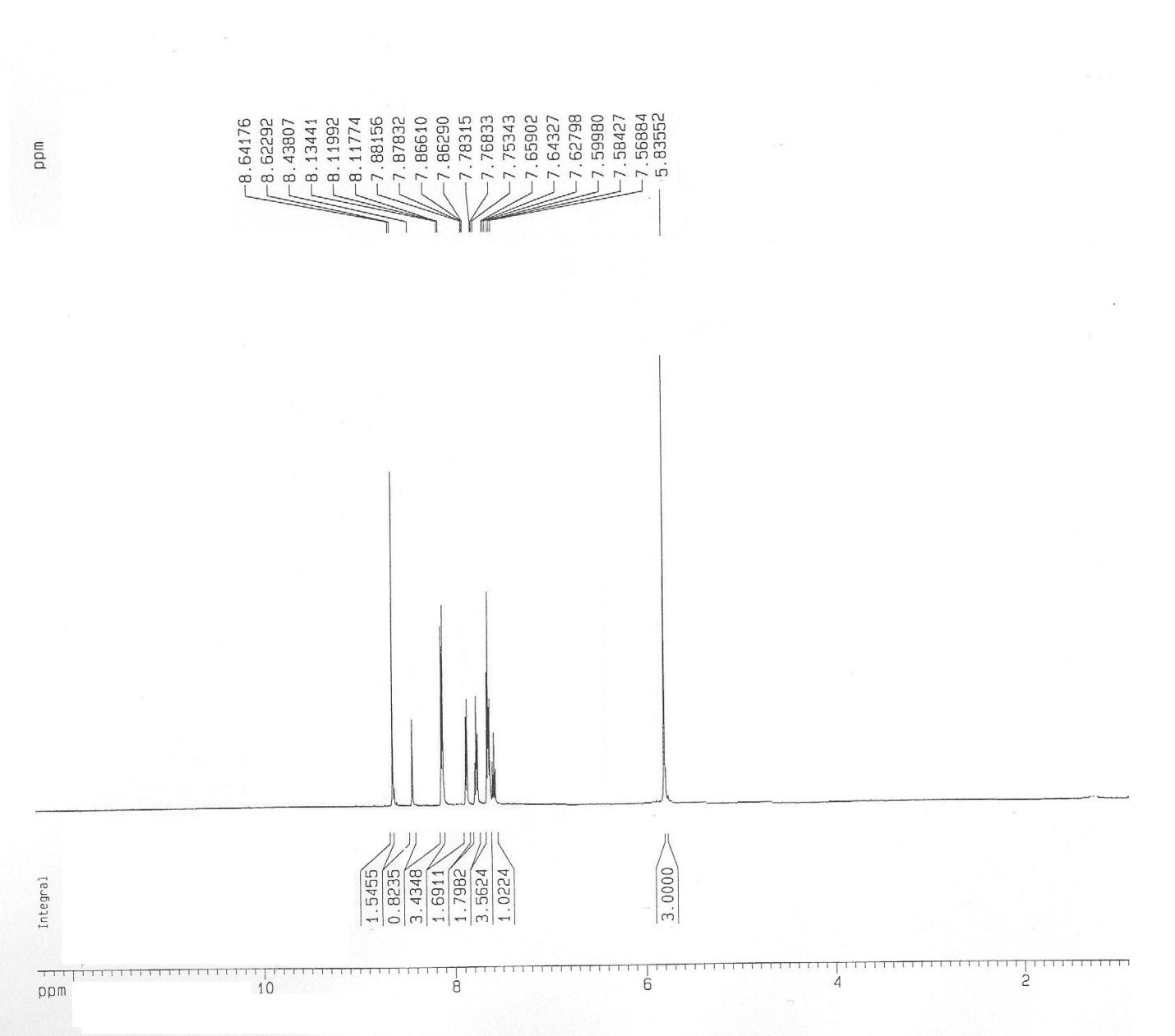

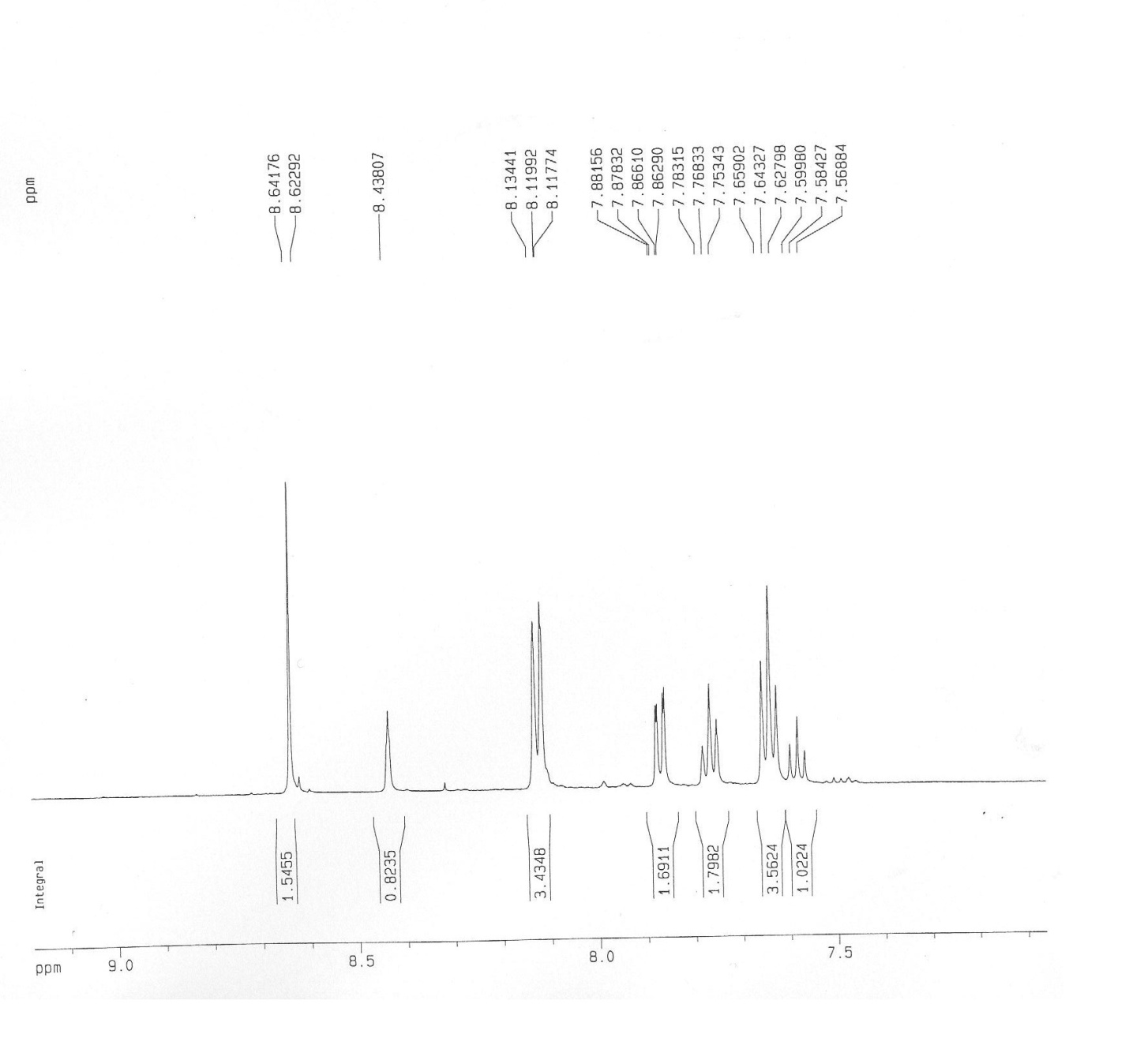

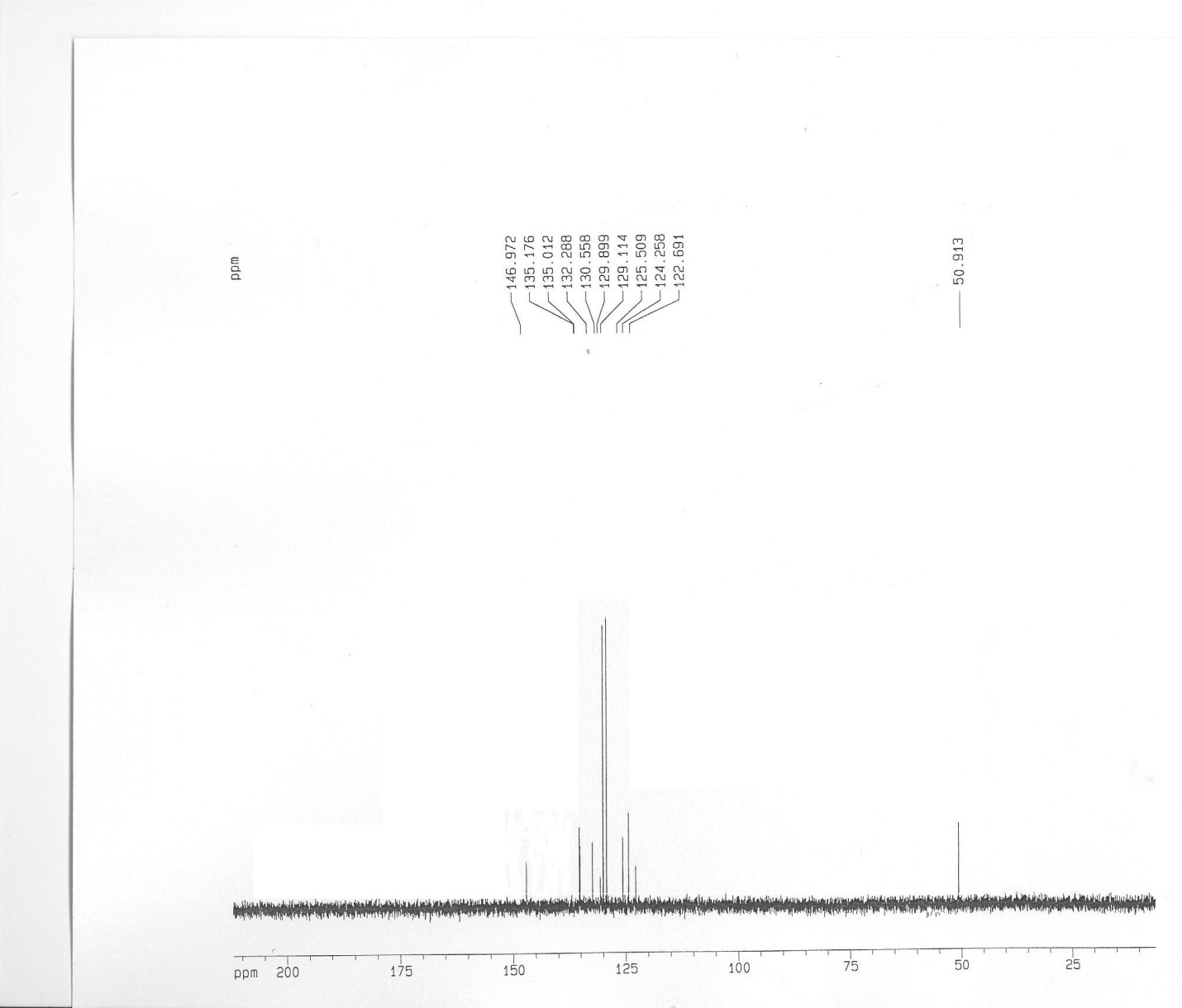

Supplement: Supplementary file 1 — Additional file 1: SEM image of Amberlyst supported nanoparticles of CuI. XRD spectrum of Amberlyst supported nanoparticles of CuI. IR spectrum of amberlite supported azide (IRA-400N3). (DOCX 4 MB) [file 40064_2012_222_MOESM1_ESM.docx]
